# Effect of peer health education intervention on HIV/AIDS knowledge amongst in-school adolescents in secondary schools in Imo State, Nigeria

**DOI:** 10.1186/s12889-024-18536-4

**Published:** 2024-04-12

**Authors:** Chinelo Judith Ezelote, Nkechi Joy Osuoji, Adaku Joy Mbachu, Chikadibia Kizito Odinaka, Ogochukwu Mildred Okwuosa, Chinaemelum Juliet Oli, Chimburuoma Georgina Ignatius

**Affiliations:** Public Health Department, Federal University of Technology, Owerri Imo State, Nigeria

**Keywords:** Peer health education, Adolescents, HIV/AIDS, Knowledge

## Abstract

**Background:**

Peer education is an approach to health promotion in which community members are supported to promote health-enhancing change among their peers. The study assessed the effect of peer health education on HIV/AIDS knowledge amongst in-school adolescents in secondary schools in Imo State.

**Methods:**

This was an intervention study carried out among 296 and 287 in-school adolescents aged 15 to 19 years attending Akwakuma Girls Secondary School and Federal Government Girls College Owerri Imo State respectively. The study was in three stages: before intervention, intervention, and after intervention. The impact of peer education was evaluated twelve weeks after intervention. Data were collected using semi-structured questionnaires. The study utilized a quasi-experimental study design. The chi-square test and McNemar’s test were used to test the hypothesis with a significance level of *p* ≤ 0.05.

**Results:**

The result from the study revealed that the majority (73%) of the respondents at Akwakuma Girls Secondary School (test group) had poor knowledge of HIV/AIDS mode of transmission and prevention at baseline. The overall good knowledge of respondents in the test group improved from 27 to 81% after the intervention. 36% of the respondents in the control group had good knowledge at baseline, the knowledge of 64% of them with poor knowledge at baseline were compared post-test to those in the test group who also had poor knowledge at baseline. The knowledge of only 27.7% of those in the control group increased post-test while the remaining 72.3% still had poor knowledge. The result of the inter-school comparison using Chi-square revealed that the *p*-value was statistically significant. Intra-school comparison using McNemar’s test revealed a statistical significance for all questions in the test group, while none was positively significant in the control group.

**Conclusions:**

Peer health education improved the knowledge of the students at Akwakuma Girls Secondary School which was very low at the baseline. The knowledge of the students in the control group with poor knowledge at baseline didn’t increase post-study. Peer health education should be strengthened and expanded as one of the tools for behavior change among adolescents. There should be more focus on adolescents for HIV-targeted prevention.

**Supplementary Information:**

The online version contains supplementary material available at 10.1186/s12889-024-18536-4.

## Background

Human Immunodeficiency Virus (HIV) and Acquired Immune Deficiency Syndrome (AIDS) are among the most complex health problems of the twenty-first century [1]. HIV/AIDS infection remains a historic public health issue globally, especially in low and middle-income countries like Nigeria where access to HIV/AIDS education and the use of Voluntary Counseling and Testing (VCT) is low [2]. The Human Immunodeficiency Virus (HIV) is a virus that targets the immune system and weakens people’s defense against many infections and some types of cancer that people with healthy immune systems can more easily fight [3]. HIV, the virus that causes acquired immunodeficiency syndrome (AIDS), is a slow-acting retrovirus. It is transmitted by unprotected sexual intercourse, contaminated blood used for blood transfusions, needles contaminated with HIV, prenatally/ perinatally, and by breastfeeding [4]. Peer health education has been shown from past studies to be an effective tool in improving knowledge, attitude, and some preventive practices towards HIV/AIDS among in-school adolescents [5].

Peer education is a health promotion approach in which community members are supported to promote healthy changes among their peers [6]. It is the teaching or sharing of health knowledge, values, and behaviors while educating others with similar social backgrounds or life experiences [7, 8]. A peer is a person who has equal standing with another in age, background, social status, and interests [9]. It was noted that young people rarely talk to health personnel about sensitive issues because they often receive their information from peers and friends [10]. The use of peer educators for health improvement has been linked with the importance of peer influence in adolescence [11]. There is evidence that young people are more likely to seek help from informal sources of support such as friends in comparison to adults [12]. These findings showed that adolescents prefer to seek help for health-related concerns from their peers rather than adults or professionals. More often than not, they might receive the wrong information from their peers. This situation underscored the need for accurate sexual health information through a channel that will be welcoming and acceptable to them. Peer health education may help to bridge this gap. It will likely help to get young people to talk about their sexual activities and ensure that the right information is made available to them. The target population for this study was in-school adolescents who were presumed vulnerable to sexual health problems, partly due to inadequate sexual health knowledge and negative attitudes.

Adolescence is a transitional stage of physical and psychological development that generally occurs during the period from puberty to adulthood (typically corresponding to the age of majority) [13]. The World Health Organization definition officially designates an adolescent as someone between the ages of 10 and 19 [14]. Adolescents view themselves as being unique and as such immune to disease and death, with their thinking that something bad will happen to someone else, not me [15]. Young persons experience numerous physical and social changes, often making it difficult for them to know how to behave [16]. During adolescence, issues of emotional (if not physical) separation from parents arise ^16^. More than a quarter (38.10%) of Nigeria's population belongs to the age group 15–24 years old [17]. UNICEF in 2015 noted that in Nigeria, only one in every four young women aged 15–24 years (24 percent) has comprehensive knowledge of HIV prevention [18]. This rate was found to be below the average for West and Central Africa (33 percent). According to UNICEF (2023), adolescents and young people represent a growing share of people living with HIV worldwide [19]. In 2022 alone, 480,000 (255,000–760,000) young people between the ages of 10 to 24 were newly infected with HIV, of whom 140,000 (35,000–250,000) were adolescents between the ages of 10 and 19 (UNICEF, 2023)^15^. The immediate school environment still serves as a fertile ground for high-risk sexual behavior [20]. The majority of the Institutions of Higher Education (IHE) in Nigeria are situated either in rural areas or on the perimeter of urban cities. The host communities in most rural areas are likely to exert an influence on the pattern and dynamics of HIV infection at IHE in Nigeria [21]. There is growing evidence from several countries where HIV prevalence is decreasing, it is the young people who are reversing the trends [22]. There is a need to ensure they are exposed more to positive behaviors at this stage of their life.

However, information on knowledge of HIV/AIDS mode of transmission and prevention among in-school adolescents in Imo State, Nigeria is scarce if not completely absent. Therefore, the objective of the study was to assess the effect of peer health education intervention on HIV/AIDS knowledge amongst in-school adolescents in secondary schools in Imo State, Nigeria.

This will guide the design of tailor-made HIV intervention programmes for HIV/AIDS among adolescents in Southeast Nigeria. The results of this study will create awareness of the positive effects of peer health education on knowledge of HIV mode of transmission and prevention among in-school adolescents. It will also be of immense benefit to programme planners who are constantly in search of a more effective and efficient strategy for communicating sexual health information to youths.

## Methods

The study had test and control groups which comprised Akwakumma Girls Secondary School (AGSS) and Federal Government Girls College Owerri (FGGC) respectively. The two schools were randomly selected through simple random sampling without replacement. All the secondary schools in Owerri metropolis were written on a piece of paper, squeezed, and put in a ballot bag after which two were randomly picked. A quasi-experimental research study design was used, where the schools were randomly assigned to test and control groups by simple balloting. The students in the two schools were matched by their age, gender, class of study, and location of schools. The age range included in the study were all those within the age range of 14 to 19 years of age, and in SS1 to SS3 class. The schools were all female schools, they were both located in Owerri Urban, and the students were all Christians. The mean ages of the students were 17.06 and 16.82 at AGSS and FGGC respectively. The respondents at baseline were 583 in total, 296 and 287 in the test and the control group respectively. They comprised all the students in the senior class (SS1 to SS3) in both schools. The level of knowledge of the respondents on HIV/AIDS was assessed using the Aids Clinical Trials Group-18 (ACTG-18). ACTG is a well-validated instrument and was adopted from the study conducted by Reynolds et al. [23]. The questionnaire which comprised eighteen questions (18) and answer keys was attached in the Appendix. The questionnaire assessed the respondents’ level of knowledge of HIV; its mode of transmission and prevention. It was administered through a face-to-face method. The peer health educators were trained using the United Nations Population Fund’s (2005) Training of Trainers manual, and the United Nations Children Fund’s (UNICEF) Reproductive Health and HIV/AIDS prevention project’s manual for peer educators, produced for the National Youth Service Corps (NYSC) in Nigeria [24, 25]. They were adapted for this study as the training instrument for the peer educators. The knowledge of the students was assessed at baseline using the ACTG-18 questionnaire. The respondents who didn’t answer one-third (33%) of the questions correctly were classified as poor knowledge. Those with poor knowledge in the test group were selected for the intervention, while those who scored above 75% were recruited as peer health educators. Those in the control group did not receive the peer health education. They were only educated on personal hygiene and environmental sanitation three times but there was no mention of HIV during the whole interactions.

### Inclusion criteria

All secondary school students at FGGC Owerri and AGSS Owerri within the age range of 15 to 19.

Those who signed the informed consent.

### Exclusion criteria

Those students at FGGC Owerri and AGSS Owerri within the age range of 15 to 19 who did not consent to this study.

Those students at FGGC Owerri and AGSS Owerri within the age range of 15 to 19 whose parents did not consent for them to participate in this study.

Those students in SS1 to SS3 at FGGC Owerri and AGSS Owerri who were more than 19 years old.

### Data collection

Data was obtained using an adapted questionnaire. Ethical clearance was obtained before proceeding further with the research. The first meeting was at the office State Ministry of Education but we met the representative. The research was explained to her, including all the processes involved. She requested for the research proposal and asked us to return after a fortnight. During the second visit after two weeks, the researchers were granted oral permission to proceed with the research. The first visit was made to the schools which equate to an introductory visit. The researcher met the principal of the various schools, and staff, and explained the study in detail to them. They both asked for the ethical approval and research proposal which were given to them. The date for the second visit was fixed by the principals of the two schools. The principal of FGGC asked the researchers to return the next semester because they started their exam the following week. The researcher met the students at AGSS after a week of visiting the principal. The research objectives were explained to them, and all that was required of them. Their confidentiality and anonymity were well assured. The students started exams the following week, hence the research was paused. The researchers met the students at the two schools in the second week of school resumption and explained the research objectives to them. It was done twice at AGSS because it was assured the students might have forgotten about it during the holidays. Their informed consent was obtained, and the researchers proceeded to share the baseline questionnaires to them during the fourth time of visiting the school. The questionnaires were shared, filled, and collected on the spot. Four research assistants were recruited for this study. They were undergraduate female students of the Federal University of Technology Owerri. They were between the age ranges of 20 to 22 and in their final year in school. They helped to administer the questionnaire to the respondents. The principal researcher trained them twice a week for two weeks and explained the questionnaire to them. They helped the students who had any problem with comprehending any question. The ones they couldn’t answer were referred to the principal researcher. The study was carried out in three stages, namely, pre-intervention, intervention, and post-intervention stages. At the intervention stage, for the students in the test group, the recruitment and training of 30 students as peer educators (ten from each level) were carried out for two weeks. Those selected as peer educators were those with scored above 75% after the baseline assessment. Topics covered included rudiments information on HIV/AIDS, its mode of transmission and prevention, and other sexually transmitted infections. The training was in the form of lectures, motivational talks, and demonstrations using audiovisuals, posters, role plays, and practical demonstrations. After the training, the trained peer educators were provided with educational materials (such as hand bills, leaflets, posters, etc.) Meetings were held weekly by the peer educators in each of the classes where they discussed their progress and challenges with the researchers. The researchers provided them with supportive supervision. The students in the test group who passed just 1/3rd (33.3%) of the eighteen questions in the questionnaire after the baseline assessment were regarded as having poor knowledge and were those included in the intervention stage. Two hundred and fifty-one (251) students out of two hundred and ninety-six students (296) scored 33% and below, and they were the only ones included in the test group. The students that scored between 33.4% and 74% were 13, while those that scored 75% and above were 32 students. The peer health educators educated them on the correct information on HIV/AIDS about its mode of transmission and prevention. The researchers were around throughout the peer health education. They supervised the whole process to ensure that the right information was passed to the students from the peer health educators. At the post-intervention stage, the same questionnaire used in the pre-intervention stage was re-administered to only the students in the test group 12 weeks after intervention which was 2 weeks before their exam. The Statistical Package for the Social Sciences (SPSS) was used in the analysis of the data obtained from the study. Results were expressed in percentages, frequencies, tables, and charts. The chi-square test tool (*p* ≤ 0.05) and McNemar’s test were used to test the hypothesis to assess any significant change in their level of knowledge. A *p*-value < 0.05 was considered as significant.

The students in the control group were 287 at baseline. The same questionnaire was given to them and their HIV knowledge at baseline was assessed. One hundred and eighty-four students (184) correctly answered just 1/3rd (33.3%) and below of the eighteen questions in the questionnaire, and were regarded as having poor knowledge. Those that scored above 33.4% were 103 students, amongst whom 47 scored above 74% whereas 56 scored between 33.4% and 74%. Those 184 students were the ones included in the post-test. They were educated on personal hygiene and environmental sanitation three times (once a month), and there was no mention of HIV during the whole interaction. After three months, the questionnaire was re-administered to them; their response was collated, computed, and analyzed using regression analysis to assess any difference in their knowledge. The second data was collected exactly one week before their term exams.

## Results

The analysis as depicted in Table 1 contained the knowledge of the respondents at Akwakuma Girls Secondary School Owerri (the test group) pre and post-intervention. It showed that their knowledge of HIV (concept, its modes of transmission, and prevention) at baseline was abysmally low in all the 18 questions in the questionnaire which increased after peer health education intervention. Only 38(12.8%) of the respondents correctly stated that coughing and sneezing did not spread HIV during the pre-test, this increased to 183(85.1%) during the post-test. A person can get HIV by sharing a glass of water with someone who has HIV was correctly indicated as not a way of contracting HIV by 52(17.6%) of the respondents during pre-intervention while the number increased to 174(80.9%) in post-intervention. Pulling out the penis before a male ejaculate keeps a woman from getting HIV was correctly answered as false by 101(34.1%) at pre-test and increased to 189(87.9%) at post-intervention. Anal sex as one of the modes of acquiring HIV/AIDs was correctly answered pre-intervention by only 56(18.9%) of the respondents which increased to 149(69.3%) after the intervention. Few numbers of students (24%) knew that showering, or washing one’s genitals/private parts after sex cannot keep someone from getting HIV, this increased to 123(57.2%) after the intervention. Only 21.6% of the respondents knew that people who have been infected with HIV do not quickly show serious signs of being infected, their knowledge of that increased to 57.7% after the intervention. Only 3.7% of the respondents knew there is currently no vaccine that can stop adults from getting HIV, the number increased to 63.3% after the intervention. At pre-intervention, only 29(9.8%) of the adolescents disagreed that people are likely to get HIV by deep kissing, or putting their tongue in their partner has mouth if their partner has HIV while their knowledge increased to 172(80.0%) after intervention. To determine if a person can get HIV by sitting in a hot tub or swimming pool with a person who has HIV, only 25(8.4%) of the respondents answered correctly while after intervention the number of knowledgeable students increased to 176(81.9%). Very few (4.4%) of the students knew that a person could get HIV from oral sex while the number increased to 83.7% after intervention. Finally, it was found that 32(10.8%) of the respondents knew that using Vaseline or baby oil with a condom cannot lower the chance of getting HIV while after intervention 140(65.1%) of the students became aware that using Vaseline or baby oil with a condom cannot lower the chance of getting HIV.

**Table 1:** Knowledge of the students on HIV/AIDS at AGSS (Test Group)

Characteristic	Responses	Frequency	
		**Baseline (*n* = 296)**	**After three months (*n* = 215)**
Coughing and Sneezing Do Not Spread HIV (X1)	False	81(27.4%)	21(9.8%)
	True	38(12.8%)	183(85.1%)
	I don’t know	177(59.8%)	11(5.1%)
A person can get HIV by sharing a glass of water with someone who has HIV (X2)	False	52(17.6%)	174(80.9%)
	True	38(12.8%)	18(8.4%)
	I don’t know	206(69.6%)	23(10.7%)
Pulling out the penis before a male ejaculate keeps a woman from getting HIV (X3)	False	101(34.1%)	189(87.9%)
	True	14(4.7%)	21(9.8%)
	I don’t know	181(61.1%)	5(2.3%)
A woman can get HIV she if has anal sex with a man (X4)	False	87(29.4%)	45(20.9%)
	True	56(18.9%)	149(69.3%)
	I don’t know	153(51.7%)	21(9.8%)
Showering, or washing one’s genitals/private parts after sex keeps a person from getting HIV (X5)	False	71(24.0%)	123(57.2%)
	True	64(21.6%)	57(26.5%)
	I don’t know	161(54.4%)	35(16.3%)
All pregnant women infected with HIV will have babies born with AIDS (X6)	False	25(8.4%)	123(57.2%)
	True	11(3.7%)	79(36.7%)
	I don’t know	260(87.8%)	13(6.0%)
People who have been infected with HIV quickly show serious signs of been infected (X7)	False	64(21.6%)	124(57.7%)
	True	23(7.8%)	73(34.0%)
	I don’t know	209(70.6%)	18(8.4%0
There is vaccine that can stop adults from getting HIV (X8)	False	93(31.4%)	136(63.3%)
	True	11(3.7%)	52(24.2%)
	I don’t know	192(64.9) %	27(12.6%)
People are likely to get HIV by deep kissing, putting their tongue in their partner has HIV (X9)	False	120(40.5%)	172(80.0%)
	True	29(9.8%)	37(17.2%)
	I don’t know	147(49.7%)	6(2.8%)
A woman cannot get HIV if she has sex during her period (X10)	False	14(4.7%)	183(85.1%)
	True	15(5.1%)	21(9.8%)
	I don’t know	267(90.2%)	11(5.1%)
There is a female condom that can help decrease a woman’s chance of getting HIV (X11)	False	123(41.6%)	90(41.9%)
	True	19(6.4%)	107(49.8%)
	I don’t know	154(52.0%)	18(8.4%)
A natural skin condom works better against HIV than the latex condom (X12)	False	12(4.1%)	153(71.2%)
	True	38(12.8%)	45(20.9%)
	I don’t know	24	17(7.9%)
A person will not get HIV if he/she is taking antibiotics (X13)	False	11(3.7%)	154(71.6%)
	True	26(8.8%)	47(21.9%)
	I don’t know	259(87.5%)	14(6.5%)
Having sex with more than one partner can increase a person’s chance of been infected with HIV (X14)	False	66(22.3%)	28(13.0%)
	True	77(26%)	169(78.6%)
	I don’t know	153(52%)	18(8.4%)
Taking a test for HIV one a week after having unprotected sex will tell a person if he/she has HIV (X15)	False	99(33%)	183(85.0%)
	True	44(15%)	30(14.0%)
	I don’t know	153(51.7%)	2(1%)
A person can get HIV by sitting in a hot tub or swimming pool with a person who has HIV (X16)	False	25(8.4%)	176(81.9%)
	True	11(3.7%)	28(13.0%)
	I don’t know	260(87.8%)	11(5.1%)
A person can get HIV from oral sex (X17)	False	52(17.6%)	23(10.7%)
	True	13(4.4)	180(83.7%)
	I don’t know	231(78.0%)	12(5.6%)
Using Vaseline or baby oil with condom lowers the chance of getting HIV (X18)	False	32(10.8%)	140(65.1%)
	True	11(3.7%)	56(26.0%)
	I don’t know	253(85.5%)	19(8.8%0

Table 2 below indicates that the *P*-value is less than 0.05. This showed that there is a significant difference in the knowledge of the respondents before and after the test. The knowledge of the respondents increased after receiving peer-health education. This indicates that peer health education had a positive impact on the HIV/AIDS knowledge of respondents in the test group.

**Table 2:** Intra school comparison of the knowledge of the students on HIV/AIDS at AGSS using McNemar's test

	**Pre-test (296)**	**Post-test(215)**	*P*-value	C.I(95%)
Good knowledge of HIV/AIDS	81 (27%)	174(81%)	.000	0.044

### Knowledge of the students on HIV/AIDS at FGGC Owerri (Control Group)

The study evaluated the knowledge of students in the control group (FGGC Owerri) before and after an intervention. The findings are presented in Table 3 below. At the start of the study, the students had a fair understanding of HIV transmission and prevention. However, 184 students who scored 33.3% or less were assessed in a post-test, and the results showed that their knowledge had not improved.

**Table 3:** Knowledge of the students on HIV/AIDS at FGGC Owerri (Control Group)

Characteristic	Responses	Frequency	
		**Baseline 287**	**After three months 184**
Coughing and Sneezing Do Not Spread HIV	True	70(24.4%)	22(12%)
	False	114(39.7%)	71(38..5%)
	I don’t know	103(35.9%)	91(49.5%)
A person can get HIV by sharing a glass of water with someone who has HIV	True	69(24.0%)	94(51.1%)
	False	123(42.9%)	34 (18.5%)
	I don’t know	95(33.1%)	56(30.4%)
Pulling out the penis before a male ejaculate keeps a woman from getting HIV	True	68(23.7%)	72(39.1%)
	False	126(43.9%)	38(20.7%)
	I don’t know	93(32.4%)	74(40.2%)
A woman can get HIV she if has anal sex with a man	True	61(21.3%)	33(18%)
	False	134(46.7%)	71(39%)
	I don’t know	92(32.0%)	80(43%)
Showering, or washing one’s genitals/private parts after sex keeps a person from getting HIV	True	96(33.4%)	72(39.1%)
	False	101(35.2%)	65 (35.3%)
	I don’t know	90(31.4%)	47(25.5%)
All pregnant women infected with HIV will have babies born with AIDS	True	71(24.7%)	47(25.5%)
	False	136(47.4%)	54(29.3%)
	I don’t know	80(27.9%)	83(45.1%)
People who have been infected with HIV quickly show serious signs of been infected	True	68(23.7%)	53(28.8%)
	False	120(41.8%)	42 (22.8%)
	I don’t know	99(34.5%)	89(48.4%)
There is vaccine that can stop adults from getting HIV	True	61(21.3%)	51(27.7%)
	False	128(44.6%)	48(26.1%)
	I don’t know	98(34.1%)	85(46.2%)
People are likely to get HIV by deep kissing, putting their tongue in their partner has HIV	True	77(26.8%)	81(44%)
	False	111(38.7%)	24(13%)
	I don’t know	99(34.5%)	80(43%)
A woman cannot get HIV if she has sex during her period	True	78(27.2%)	69(37.5%)
	False	115(40.0%)	44(23.9%)
	I don’t know	94(32.8%)	71(38.6%)
There is a female condom that can help decrease a woman’s chance of getting HIV	True	87(30.3%)	41(22.3%)
	False	111(38.7%)	44(23.9%)
	I don’t know	89(31.0%)	99(53.8%)
A natural skin condom works better against HIV than the latex condom	True	84(29.3%)	48(26.1%)
	False	94(32.7)	42(22.8%)
	I don’t know	109(38.0)	94(51.1%)
A person will not get HIV if he/she is taking antibiotics	True	92(32.1%)	72(39.1%)
	False	110(38.3%)	40(21.7%)
	I don’t know	85(29.6%)	72(39.1%)
Having sex with more than one partner can increase a person’s chance of been infected with HIV	True	124(43.2%)	61(33.2%)
	False	65(23%)	40(21.7%)
	I don’t know	98(34%)	83(45.1%)
Taking a test for HIV one a week after having unprotected sex will tell a person if he/she has HIV	True	73(25.4%)	55(29.9%)
	False	137(47.7%)	50(27.2%)
	I don’t know	77(26.9%)	79(42.9%)
A person can get HIV by sitting in a hot tub or swimming pool with a person who has HIV	True	58(20.2%)	48(26.1%)
	False	137(47.7%)	46(25%)
	I don’t know	92(32.1%)	90(48.9%)
A person can get HIV from oral sex	True	103(35.9%)	39(21.2%)
	False	90(31.4%)	43(23.4%)
	I don’t know	94(32.8%)	102(55.4%)
Using Vaseline or baby oil with condom lowers the chance of getting HIV	True	89(31.0%)	55(29.9%)
	False	125(43.6%)	53(28.8%)
	I don’t know	73(25.4%)	76(41.3%)

### Intra school comparison of the knowledge of the students on HIV/AIDS at FGGC Owerri using Regression analysis

At the post-test, respondents with poor knowledge at the beginning (≤ 33.3%) were re-assessed without any intervention to determine if their knowledge increased. The results, as presented in Table 4, showed that only 27.7% of the respondents had increased knowledge, while the remaining 73.3% still had poor knowledge at the post-test. The *P*-value was 0.05, indicating a significant difference in the knowledge levels between the pre and post-test. The good knowledge level at baseline was higher compared to the post-test.

**Table 4:** Intra school comparison at FGGC Owerri using McNemar’s test

	**Pre-test (287)**	**Post-test(184)**	***P*-value**	**C.I(95%)**
Good knowledge of HIV/AIDS	103(36%)	51(27.7%)	0.05	1.145

### Overall knowledge of the respondents at pre and post test

Table 5 below shows the overall knowledge level of the respondents before and after the test. At the beginning of the study, 85% of the respondents at AGSS had poor knowledge (less than 33.3%). However, by the end of the study, this percentage decreased to 30.6. In the control group, 64.1% of the respondents had poor knowledge at baseline. Among those who had poor knowledge (< 33.3%) in the control group at the beginning of the study, 72.3% still had poor knowledge of HIV at the end of the study, while 27.7% showed an increase in knowledge (from < 33.3% to ≥ 33.4 and above).

**Table 5:** Overall knowledge of the respondents at pre and post test

**school**		**Overall knowledge**	
		**Pre-test (FGGC *n* = 287)** **(AGSS *n* = 296)**	**Post Test (FGGC *n* = 184)** **(AGSS *n* = 215)**
**FGGC**	< 33.3%	184(64.1%)	133 (72.3%)
	33.4%- 74.9%	56(19.5%)	48 (26.1%)
	75% and above	47(16.4%)	3 (1.6%)
	**Total**	**287 (100%)**	**184 (100%)**
**AGSS**	< 33.3%	251(85%)	77 (30.6%)
	33.4%- 74.9%	13(4%)	92 (36.7%)
	75% and above	32(11%	82(32.7%)
	**Total**	**296 (100%)**	**251 (100%)**

### Comparison of the respondents in the control group’s knowledge pre and post-intervention

An intra-school comparison was conducted to determine if there was a significant difference in students' knowledge of HIV/AIDS between two secondary schools before and after an intervention. A chi-square test of association was performed, and the results are presented in Table 6. The *p*-value was less than 0.05 (95% confidence level) for all variables except X13 and X16. This indicates that there was a significant difference in knowledge levels between the test group (who received the intervention) and the control group at both the pre-test and post-test stages.

**Table 6:** Chi square analysis of the good and poor knowledge of the students on HIV/AIDS at FGGC and AGSS Owerri (at baseline and post-intervention)

Characteristic	SCHOOL	Good knowledge		Poor knowledge		df	X^2^	*P*-Value
		**Pre-test** **(FGGC *n* = 287)** **(AGSS *n* = 296)**	**Post Test** **(FGGC *n* = 184)** **(AGSS *n* = 215)**	**Pre-test** **(FGGC *n* = 287)** **(AGSS *n* = 296)**	**Post Test** **(FGGC *n* = 184)** **(AGSS *n* = 215)**			
Coughing and Sneezing Do Not Spread HIV(X1)	FGGC	70(24.4%)	22(12%)	217(75.6%)	162(88%)	1	63.482	0.000
	AGSS	38(12.8)	183(85.1%)	258(87.2%)	32(14.9%)			
X2	**FGGC**	123(42.9%)	34 (18.5%)	164(57.1%)	150(81.5%)	1	12.226	0.000
	**AGSS**	52(17.6%)	174(80.9%)	244(82.4%)	41(19.1%)			
X3	**FGGC**	126(43.9%)	38(20.7%)	161(56.1)	146(79.3%)	1	47.426	0.000
	**AGSS**	101(34.1%)	189(87.9%)	195(65.9%)	26(12.1%)			
X4	**FGGC**	61(21.3%)	33(18%)	226(78.3%)	151(82%)	1	47.037	0.000
	**AGSS**	56(18.9%)	149(69.3%)	240(81.1%)	66(30.7%)			
X5	**FGGC & AGSS**	101(35.2%)	65 (35.3%)	186(64.8)	119(64.7%)	1	0.781	0.036
		71(24.0%)	123(57.2%)	225(76%)	92(42.8%)			
X6	**FGGC**	136(47.4%)	54(29.3%)	151(52.6%)	130(70.7%)	1	14.054	0.000
	**AGSS**	25(8.4%)	123(57.2%)	271(91.6%)	92(42.8%)			
X7	**FGGC**	120(41.8%)	42 (22.8%)	167(58.2%)	142(77.2%)	1	.688	0.038
	**AGSS**	64(21.6%)	124(57.7%)	232(78.4%)	89(42.3%)			
X8	**FGGC**	128(44.6%)	48(26.1%)	159(55.4%)	136(73.9%)	1	5.609	0.003
	**AGSS**	93(31.4%)	136(63.3%)	203(69.6%)	79(36.7%)			
X9	**FGGC**	111(38.7%)	24(13%)	176(61.3%)	160(87%)	1	80.896	0.000
	**AGSS**	120(40.5%)	172(80.0%)	176(59.5%)	43(20%)			
X10	**FGGC**	115(40.0%)	44(23.9%)	172(60%)	140(76.1%)	1	2.437	0.016
	**AGSS**	14(4.7%)	183(85.1%)	282(95.3%)	32(14.9%)			
X11	**FGGC**	87(30.3%)	41(22.3%)	200(69.7%)	143(77.7%)	1	.811	0.039
	**AGSS**	19(6.4%)	107(49.8%)	277(93.6%)	108(50.2%)			
X12	**FGGC**	94(32.7%)	42(22.8%)	193(67.2%)	142(77.2%)	1	59.625	0.000
	**AGSS**	12(4.1%)	153(71.2%)	62(20.9%)	62(28.8%)			
X13	**FGGC**	110(38.3%)	40(21.7%)	177(61.7%)	144(78.3%)	1	.022	0.054
	**AGSS**	11(3.7%)	154(71.6%)	285(96.3%)	61(28.4%)			
X14	**FGGC**	124(43.2%)	61(33.2%)	163(56.8%)	123(66.8%)	1	7.817	0.001
	**AGSS**	77(26%)	169(78.6%)	219(74%)	46(21.4%)			
X15	**FGGC**	137(47.7%)	50(27.2%)	150(52.3%)	134(72.8%)	1	23.550	0.000
	**AGSS**	99(33%)	183(85.0%)	197(67%)	32(15%)			
X16	**FGGC**	137(47.7%)	46(25%)	150(52.3%)	138(75%)	1	.024	0.052
	**AGSS**	25(8.4%)	176(81.9%)	271(91.6%)	39(18.1%)			
X17	**FGGC**	103(35.9%)	39(21.2%)	184(64.1%)	145(78.8%)	1	6.332	.002
	**AGSS**	13(4.4%)	180(83.7%)	283(95.6%)	35(16.3%)			
X18	**FGGC**	125(43.6%)	53(28.8%)	162(56.4%)	131(71.2%)	1	1.825	.021
	**AGSS**	32(10.8%)	140(65.1%)	264(89.2%)	75(34.9%)			

### Chi square for the overall knowledge test of association

The overall knowledge of the respondents in the two schools was compared using the Chi-square test and presented in Table 7 below. They were grouped into three categories; those that scored ≥ 33.3%, those that scored between 33.4% – 74.9%, and those that scored ≥ 75%. The test results showed the values for all the groups were less than 0.05. There is a statistical difference in the knowledge of the respondents pre and post-test for all the categories.

**Table 7:** CHI SQUARE for the overall knowledge test of association

School	Percentage response	Overall knowledge	
		**Pre-test** **(FGGC *n* = 287)** **(AGSS *n* = 296)**	**Post Test** **(FGGC *n* = 184)** **(AGSS *n* = 215)**
**FGGC**	< 33.3%	184(64.1%)	133 (72.3%)
**AGSS**	< 33.3%	251(85%)	77 (30.6%)
***P* value**	.013^a^		
**FGGC**	33.4%- 74.9%	56(19.5%)	48 (26.1%)
**AGSS**	33.4%- 74.9%	13(4%)	92 (36.7%)
***P* Value**	.002^a^		
**FGGC**	75% and above	47(16.4%)	3 (1.6%)
**AGSS**	75% and above	32(11%	82(32.7%)
***P* value**	.000^a^		

### Summary of the tables

Table 8 below depicts the summary of all the tables. The Chi-square analysis showed that the knowledge of the respondents was statistically significant pre and post-test.

**Table 8:** Summary of the tables

	**Schools**	**Pre-test** **(FGGC *n* = 287)** **(AGSS *n* = 296)**	**Post Test** **(FGGC *n* = 184)** **(AGSS *n* = 215)**	*P* value
Good Knowledge Of HIV/AIDS	AGSS	81 (27%)	174(81%)	.000
	FGGC	103(36%)	51(28%)	
Poor Knowledge Of HIV/AIDS	AGSS	215(73%)	41(19%)	.000^a^
	FGGC	184(64%)	133(72%)	

## Discussion

### Knowledge of HIV/AIDS among in-school adolescents at baseline

The result of this study as shown in Table 1 revealed that the knowledge of HIV/AIDS (concept, mode of transmission, and prevention) among in-school adolescents in Akwakuma Girls Secondary School Imo State was abysmally low at baseline, with only 27% of the students knowing about HIV/AIDS at baseline. They had low knowledge of all the eighteen questions in the questionnaire, while students in the control group had better knowledge of HIV compared to those in the test group at baseline. This is consistent with the study on HIV comprehensive knowledge and prevalence among 1818 young adolescents in Akwa Ibom State Nigeria using the AIDS indicator survey, 2017 [26]. The result of the study showed low levels of comprehensive HIV knowledge (9.4%) among young adolescents, and the majority (93%) of young adolescents perceived themselves not to be at risk of HIV. Another study on peer education as an effective behavior change strategy among in-school adolescents attending mixed secondary school in Osun State using a pretested semi-structured questionnaire, revealed that although the level of awareness about AIDS at the pre-intervention stage was very high with more than 9 out of 10 respondents in both the study and control groups being aware of the disease called AIDS, the comprehensive knowledge about HIV/AIDS was rather poor [27].

### Knowledge of HIV/AIDS among in-school adolescents after Intervention

The findings showed that the knowledge of HIV/AIDS among the respondents in the test group rapidly increased after the peer-based health education intervention was conducted. From the result presented in Table 1, the knowledge of the respondents in the test group increased for all the questions after the study intervention. Their knowledge increased from 27 to 81%. The result of the Chi-square test analysis comparing the students’ knowledge of HIV at baseline and after study intervention showed that the test was statistically significant for all the eighteen questions (*P* < 0.05) except questions 13 and 16. This means that the knowledge of the students in the test group increased for almost all the questions after the study intervention. The knowledge of the majority (73.3%) of the respondents in the control group which scored 33.3% at baseline didn’t increase for any of the questions at the post-test. This result was in line with an Intervention study conducted by Adeomi et al. which was conducted in three stages; before intervention, intervention, and after intervention^23^. The impact of peer education was evaluated 12 weeks after intervention. After the peer education intervention, those with good knowledge and positive attitudes towards HIV/AIDS increased significantly from 50.0% to 86.7% and from 49.0% to 85.6% respectively (*P* < 0.05). This finding is also consistent with the findings of Chizoba A.F et al. on the effects of peer and provider-based education interventions on HIV/AIDS knowledge and behaviour risk among in-school adolescents in Nigeria [28]. The researchers noted very significant differences between intervention groups and control groups after intervention. The study conducted in the Dominican Republic reported that respondents who received sex education (intervention group) were 1.72 times more likely to have high HIV/AIDS knowledge than respondents who reported not receiving sex education (control group) [29]. A program evaluation study of developing countries similarly demonstrated that participants who received HIV prevention education intervention reported superior knowledge when compared with the control group. The study conducted to assess the effects of peer education on AIDS knowledge and sexual behavior among youths on national service and secondary school students in Nigeria further showed that both youths and students who received HIV (prevention intervention) HPI reported superior knowledge of HIV/AIDS than their counterparts who did not. The result showed that the peer health education intervention had positive effects on both youths and students who received the intervention [30]. A study conducted on the Impact of a Peer Public Health Education Programme on Adolescent Students’ Knowledge of HIV/AIDS and Attitude Towards People Living with HIV/AIDS in Abia State, South East Nigeria revealed that the adolescent students who were given peer education training attained higher knowledge of HIV/AIDS and also showed a greater positive attitude towards people living with HIV/AIDS. The researchers noted that the result of the research proved that peer education training is evidenced in attaining higher knowledge of HIV/AIDS and in showing a greater positive attitude towards people living with HIV/AIDS [31]. Also, the study conducted in Khartoum, Suda on the effect of AIDS peer health education on knowledge, attitudes, and practices of secondary school students showed that the intervention program improved participants’ knowledge from 75.5% to 83.2%. The study concluded that school peer education is an effective approach to inform students of unsafe sexual behavior about HIV/AIDS [32].

Peer-health education is encouraged to be used as a means of improving HIV/AIDS knowledge/awareness among adolescents and young adults to achieve HIV pandemic control especially as adolescents/young adults are contributing to more than half of new infections.

## Conclusion

This study showed that the knowledge of in-school adolescents in the Akwakumma Girls Secondary School Owerri in Imo State was low at the baseline. Baseline HIV knowledge among the adolescents was unimpressive, and this calls for urgent concern. Peer-based health education resulted in better knowledge of the students in the test group on information on HIV/AIDS; its mode of transmission and prevention. The knowledge of the students in the control group did not improve during the post-test. Adolescents are the leaders of the next generation hence they need to be adequately equipped with the right information on HIV/AIDS for targeted prevention.

### Recommendation

It is recommended that this same study should be replicated in many areas, and peer-based health education should be inculcated in the curricula of secondary schools. This will ensure they are getting the right information. They are at the stage where they have access to much information, hence the need to ensure they are getting the right ones.

### Supplementary Information

**Supplementary Material 1.**

## Data Availability

All data generated or analyzed during this study are included in this published article.
