# Supplementary material for: Effect of peer health education intervention on HIV/AIDS knowledge amongst in-school adolescents in secondary schools in Imo State, Nigeria
Source: BMC Public Health. 2024 Apr 12;24:1029. doi: 10.1186/s12889-024-18536-4 (PMC11015591; doi:10.1186/s12889-024-18536-4)
Supplement: Supplementary file 1 — Supplementary Material 1. [file 12889_2024_18536_MOESM1_ESM.docx]

**HIV-KQ-18**

**For each statement, please circle “True” (T), “False” (F), or “I don’t know” (DK). If you do not know, please do not guess; instead, please circle “DK.”**

| **True False I don’t**  **know** |
| --- |

X1 Coughing and sneezing DO NOT spread HIV. T F DK

X2. A person can get HIV by sharing a glass of water with
someone who has HIV. T F DK

X3. Pulling out the penis before a man climaxes/cums keeps
a woman from getting HIV during sex. T F DK

X4. A woman can get HIV if she has anal sex with a man. T F DK

X5. Showering, or washing one’s genitals/private parts,
after sex keeps a person from getting HIV. T F DK

X6. All pregnant women infected with HIV will have babies
born with AIDS. T F DK

X7. People who have been infected with HIV quickly show
serious signs of being infected. T F DK

X8. There is a vaccine that can stop adults from getting HIV. T F DK

X9.People are likely to get HIV by deep kissing, putting their
tongue in their partner’s mouth, if their partner has HIV. T F DK

X10.A woman cannot get HIV if she has sex during her period. T F DK

X11. There is a female condom that can help decrease a woman’s
chance of getting HIV. T F DK

X12. A natural skin condom works better against HIV than does
a latex condom. T F DK

X13. A person will NOT get HIV if she or he is taking antibiotics. T F DK

X14. Having sex with more than one partner can increase a
person’s chance of being infected with HIV. T F DK

X15. Taking a test for HIV one week after having sex will tell a
person if she or he has HIV. T F DK

X16.A person can get HIV by sitting in a hot tub or a swimming
pool with a person who has HIV. T F DK

X17. A person can get HIV from oral sex. T F DK

X18. Using Vaseline or baby oil with condoms lowers the
chance of getting HIV. T F DK

Answer Key
HIV KQ 18

| **X1** | TRUE |
| --- | --- |
| **X2** | FALSE |
| **X3** | FALSE |
| **X4** | TRUE |
| **X5** | FALSE |
| **X6** | FALSE |
| **X7** | FALSE |
| **X8** | FALSE |
| **X9** | FALSE |
| **X10** | FALSE |
| **X11** | TRUE |
| **X12** | FALSE |
| **X13** | FALSE |
| **X14** | TRUE |
| **X15** | FALSE |
| **X16** | FALSE |
| **X17** | TRUE |
| **X18** | FALSE |
